# Supplementary material for: The Role of Ebola Virus VP24 Nuclear Trafficking Signals in Infectious Particle Production
Source: bioRxiv. 2024 Mar 13:2024.03.13.584761. Preprint. [Version 1] doi: 10.1101/2024.03.13.584761 (PMC10980025; doi:10.1101/2024.03.13.584761)
Supplement: 1 [file NIHPP2024.03.13.584761v1-supplement-1.pdf]

bioRxiv preprint doi: https://doi.org/10.1101/2024.03.13.584761; this version posted March 13, 2024. The copyright holder for this preprint (which was not certified by peer review) is the author/funder, who has granted bioRxiv a license to display the preprint in perpetuity. It is made available under aCC-BY 4.0 International license.

Supporting Information Captions

S1 Figure: Viral Genome Replication and trVLP Production are Reduced for VP24 Mutant trVLPs

A-B) Quantification of viral RNA at P1. P0 trVLP supernatants generated from the indicated minigenomes were harvested and used to infect transfected target cells at P1. –L indicates a P0 transfection with the VP24 WT minigenome in which the L expression plasmid was omitted. Seventy-two hours post-infection, cells were harvested for RNA extraction and cDNA was generated using random hexamer as primer. Reverse transcription-quantitative PCR (RT-qPCR) was performed using primers targeting VP40 (A) or the EBOV 5' trailer (B), as described previously [51]. Data are represented as fold expression relative to the -L control. **** denotes p-value ≤ 0.001. *** denotes p-value ≤ 0.001. C) Western blot analysis of viral protein expression in trVLPs from P0. Following a P0 transfection in Huh7 cells, trVLP supernatants were concentrated by ultracentrifugation through a sucrose cushion. The concentrated trVLPs were analyzed by western blot for viral protein expression. The remaining P0 transfected cells were lysed

bioRxiv preprint doi: https://doi.org/10.1101/2024.03.13.584761; this version posted March 13, 2024. The copyright holder for this preprint (which was not certified by peer review) is the author/funder, who has granted bioRxiv a license to display the preprint in perpetuity. It is made available under aCC-BY 4.0 International license.

and the whole cell extracts (WCE) were analyzed by Western blot for viral protein expression.

S2 Figure: VP24-WT, VP24-1A, VP24-NES, and VP24-1A+NES Colocalize with Viral Inclusion Bodies

A-B) Confocal microscopy images of P0 transfected Huh7 cells. Seventy-two hours post-transfection with the indicated minigenomes, cells were fixed and stained for VP24, VP35, and nuclei. Confocal microscopy images were captured using a Biotek Cytation C10 at 60x.

S3 Figure: Alignment of VP24 WT, VP24-ENSS, and VP24-PKI NES C-termini

An alignment of the 240-251 C-terminal residues for the VP24 WT, VP24-ENSS, and VP24-PKI NES. Yellow highlighted residues indicate the VP24 WT NES. Residues in red indicate residues that differ from the VP24 WT sequence. The hyphen was inserted into the PKI NES to align the C-terminal most L and I with those in the other sequences.

Fig. S1

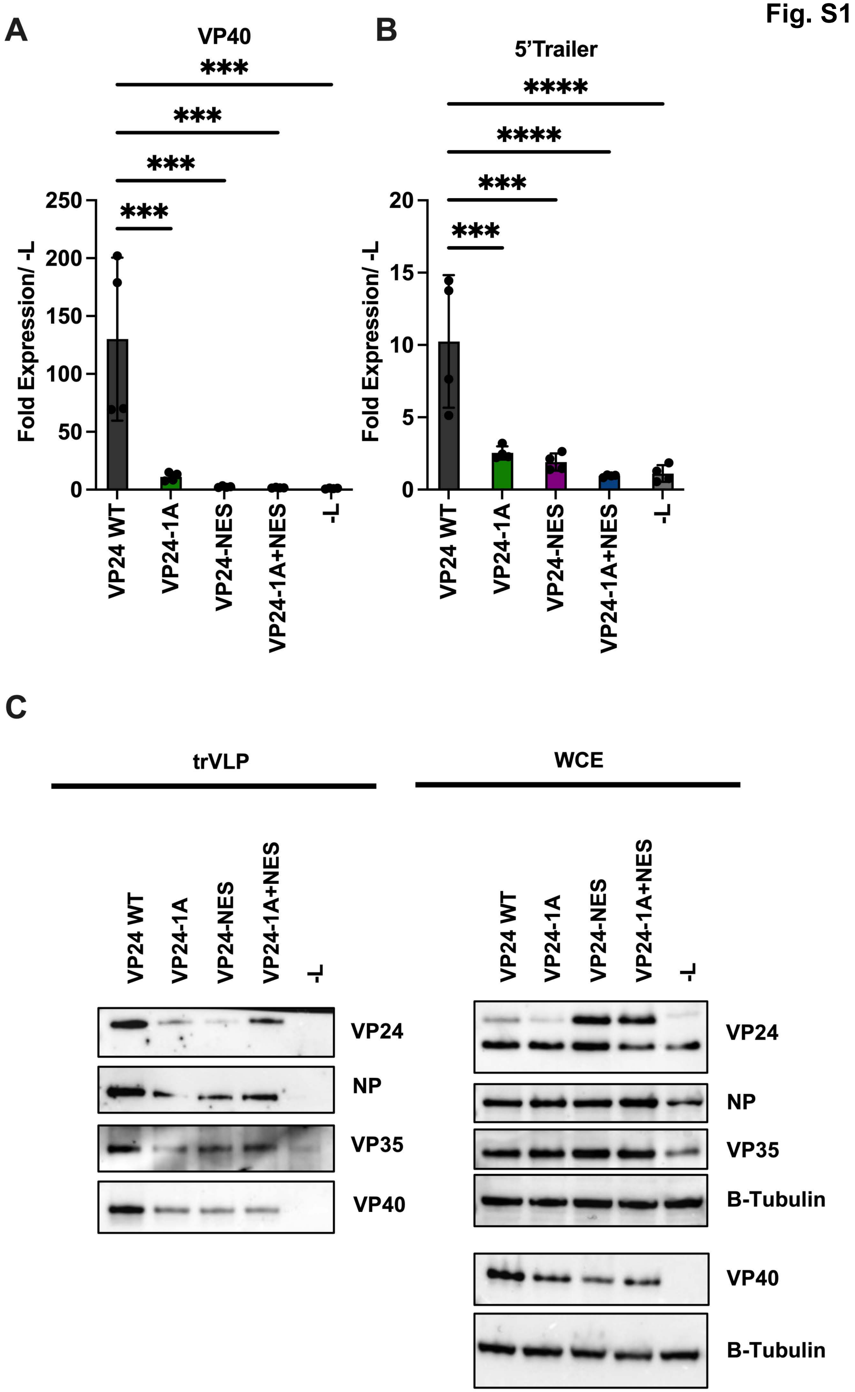
A
VP40
***
***
***
***
Fold Expression/ -L
250
200
150
100
50
0
VP24 WT
VP24-1A
VP24-NES
VP24-1A+NES
-L
B
5'Trailer
****
****
***
***
Fold Expression/ -L
20
15
10
5
0
VP24 WT
VP24-1A
VP24-NES
VP24-1A+NES
-L
C
trVLP
WCE
VP24 WT
VP24-1A
VP24-NES
VP24-1A+NES
-L
VP24
NP
VP35
VP40
VP24
NP
VP35
B-Tubulin
VP40
B-Tubulin

Fig. S2

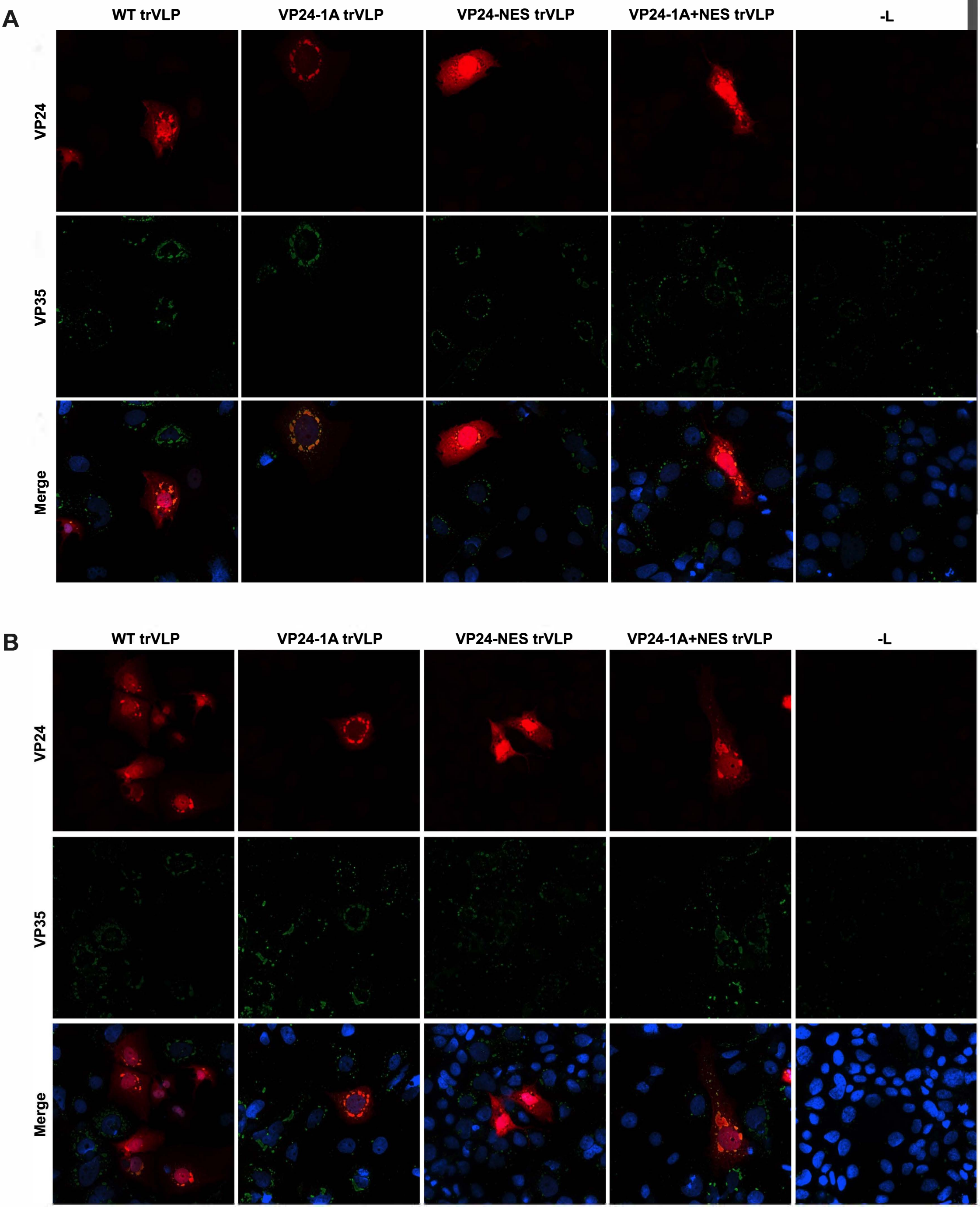
A
WT trVLP
VP24-1A trVLP
VP24-NES trVLP
VP24-1A+NES trVLP
-L
VP24
VP35
Merge
B
WT trVLP
VP24-1A trVLP
VP24-NES trVLP
VP24-1A+NES trVLP
-L
VP24
VP35
Merge

Fig. S3

**VP24 WT**	240	SLILEFNSSLAI	251
**VP24-ENSS**	240	SLILAFAAALAI	251
**VP24-PKI NES**	240	SLALKLAG-LDI	250
